# Expression of the Calcium-Binding Protein CALB1 Is Induced and Controls Intracellular Ca^2+^ Levels in Senescent Cells

**DOI:** 10.3390/ijms23169376

**Published:** 2022-08-19

**Authors:** Clotilde Raynard, Nolwenn Tessier, Anda Huna, Marine Warnier, Jean-Michel Flaman, Fabien Van Coppenolle, Sylvie Ducreux, Nadine Martin, David Bernard

**Affiliations:** 1 Centre de Recherche en Cancérologie de Lyon, Inserm U1052, CNRS UMR 5286, Centre Léon Bérard, Université de Lyon, 69373 Lyon, France; 2 CarMeN Laboratory, INSERM, INRA, INSA Lyon, Université Claude Bernard Lyon 1, 69500 Bron, France

**Keywords:** cellular senescence, Ca^2+^, CALB1, NFAT

## Abstract

In response to many stresses, such as oncogene activation or DNA damage, cells can enter cellular senescence, a state of proliferation arrest accompanied by a senescence-associated secretory phenotype (SASP). Cellular senescence plays a key role in many physiopathological contexts, including cancer, aging and aging-associated diseases, therefore, it is critical to understand how senescence is regulated. Calcium ions (Ca^2+^) recently emerged as pivotal regulators of cellular senescence. However, how Ca^2+^ levels are controlled during this process is barely known. Here, we report that intracellular Ca^2+^ contents increase in response to many senescence inducers in immortalized human mammary epithelial cells (HMECs) and that expression of calbindin 1 (CALB1), a Ca^2+^-binding protein, is upregulated in this context, through the Ca^2+^-dependent calcineurin/NFAT pathway. We further show that overexpression of CALB1 buffers the rise in intracellular Ca^2+^ levels observed in senescent cells. Finally, we suggest that increased expression of Ca^2+^-binding proteins calbindins is a frequent mark of senescent cells. This work thus supports that, together with Ca^2+^channels, Ca^2+^-binding proteins modulate Ca^2+^ levels and flux during cellular senescence. This opens potential avenues of research to better understand the role of Ca^2+^ and of Ca^2+^-binding proteins in regulating cellular senescence.

## 1. Introduction

Cellular senescence is a cell response to many stresses, such as oncogene activation, oxidative stress, telomere shortening, or DNA damage. This response is mainly characterized by a stable cell proliferation arrest and a senescence-associated secretory phenotype (SASP), consisting in the secretion of multiple factors, including, among others, pro-inflammatory cytokines and chemokines, metalloproteases and growth factors. The SASP can activate the immune system to eliminate senescent cells. Senescence is thus a critical barrier against abnormal cell proliferation and tumor formation. However, over time and upon chronic exposure to stresses, senescent cells accumulate and contribute to tumor progression through their SASP, by promoting stemness, epithelial-mesenchymal transition, and migration. Accumulation of senescent cells also critically promotes aging and aging-associated diseases. Even though some molecular pathways are already well known to regulate senescence, such as the p53/p21^CIP1^ or p16^INK4a^/Rb axis, which implement the cell cycle arrest or the NF-κB, Notch, and mTOR pathways which control the SASP, how senescence is regulated is not yet fully understood [1,2,3]. 

Calcium (Ca^2+^) is a powerful secondary messenger driving multiple cellular processes, ranging from cell proliferation and secretion to cell death. Ca^2+^ intracellular concentrations and fluxes need to be tightly controlled to achieve a fine-tune regulation of cellular responses. A pivotal role of Ca^2+^ in cellular senescence has recently emerged in the literature [4,5]. A rise in intracellular Ca^2+^ contents and activation of calcium-dependent signaling pathways, such as the calcineurin/nuclear factor of activated T cells (NFAT) pathway, have been reported in some senescence contexts. These increased contents and activity regulate senescence outcomes [6,7]. However, how intracellular Ca^2+^ levels evolve and are controlled in response to different senescence inducers, especially in epithelial cells, is barely known. 

Many proteins such as channels, transporters, or pumps can regulate Ca^2+^ transfers and levels inside the cells. A few of them have been recently described as key regulators of cellular senescence. The inositol 1,4,5-trisphosphate receptor type 2 (ITPR2), which acts as an endoplasmic reticulum (ER) Ca^2+^ release channel, is shown to promote cellular senescence and organismal aging by fostering Ca^2+^ transfers from ER to mitochondria, through mitochondria-ER contacts (MERCs) [8,9,10]. Decreased expression of transient receptor potential cation channel subfamily C member 3 (TRPC3) was observed in response to many senescence inducers. TRPC3 re-expression promotes escape from oncogene-induced senescence by decreasing mitochondrial Ca^2+^ load by inhibiting ITPR3 activity [11]. Beyond these channels, cells also have a calcium-buffering system that shapes intracellular Ca^2+^ signals. Calcium-buffering proteins are calcium-binding proteins (CaBP) that tightly control intracellular free Ca^2+^ concentrations. Indeed, they sequester free Ca^2+^ by their EF-hand domains and thus fine-tune Ca^2+^ signaling [12]. Whether calcium-buffering proteins are involved in senescence remains unknown.

In this study, we discovered that the expression of calbindin 1 (CALB1, also called calbindin-D28K), a well-known calcium-binding protein, is upregulated in response to many senescence inducers in immortalized human mammary epithelial cells (HMECs) and that CALB1 buffers the rise of Ca^2+^ observed in senescent cells. During cellular senescence in non-epithelial cells, CALB1 is not induced, but CALB2 protein might take over this Ca^2+^-buffering role.

## 2. Results

### 2.1. A Rise in Intracellular Calcium Content Is a Common Mark of Senescence in HMECT

**Figure 1:**
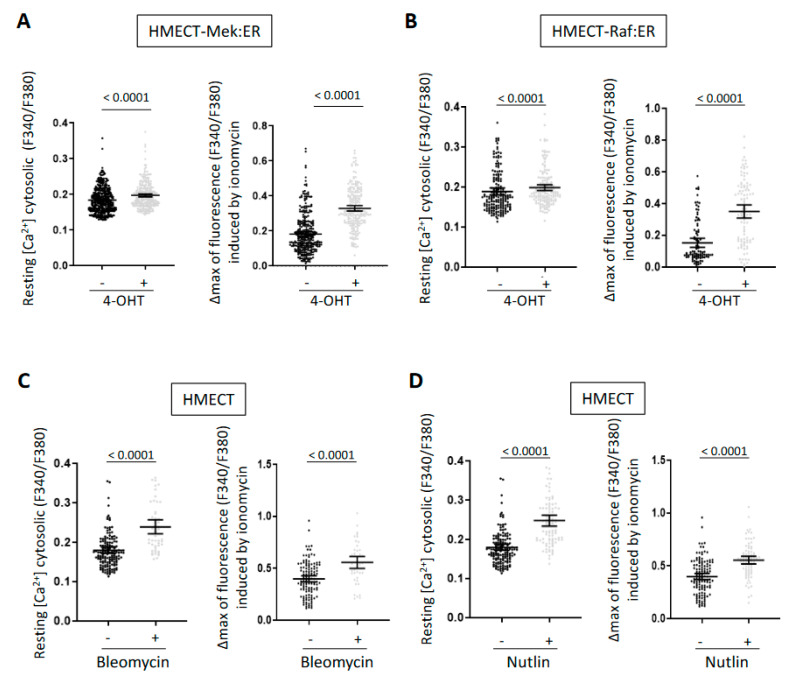
Intracellular Ca^2+^ levels are increased in HMECT in response to diverse senescence inducers. Ca^2+^ levels were measured with the ratiometric probe Fura2-AM in HMECT either overexpressing 4-OHT-inducible oncogenes Mek:ER (**A**) or Raf:ER (**B**) or treated with bleomycin (**C**) or nutlin (**D**). Resting cytosolic Ca^2+^ concentration was evaluated as stable fluorescent ratio before stimulation (**left** panels) and the size of Ca^2+^ intracellular stocks was estimated as the Ca^2+^ peak amplitude obtained after ionomycin stimulation (**right** panels). Means +/− SEM of three independent experiments are presented. Mann–Whitney *t*-test was performed, and *p*-values are indicated. Mek:ER cells: Three days after 4-OHT treatment (−4-OHT n = 391, +4-OHT n = 271), Raf:ER cells: three days after 4-OHT treatment (−4-OHT n = 173, +4-OHT n = 154), bleomycin or nutlin-treated cells: four days after treatment (non-treated n = 231, bleomycin n = 94, nutlin n = 162).

Regulation of intracellular Ca^2+^ levels has been recently reported in some contexts of cellular senescence, and we and others have demonstrated that Ca^2+^ transfer from endoplasmic reticulum to mitochondria promotes cellular senescence [8,9,10,11]. To know whether Ca^2+^ level alterations are a classical mark of senescent cells, we measured Ca^2+^ levels in HMEC in proliferation versus senescence using different types of senescence inducers: oncogenes, the chemotherapeutic agent bleomycin, or the p53 stabilizer nutlin-3. For oncogene-induced senescence (OIS), telomerase-expressing HMEC (HMECT) were infected with retroviral vectors encoding Mek or Raf oncogenes in fusion with the ligand binding domain of estrogen receptor (ER) (HMECT-Mek:ER; HMECT-Raf:ER), inducible with 4-HydroxyTamoxifen (4-OHT). These HMECT expressing inducible oncogenes enter OIS upon oncogene activation by adding 4-OHT [9,13,14,15]. Senescence was otherwise induced by the genotoxic drug bleomycin through activation of a DNA damage response or by nutlin through stabilization of p53, one of the main effectors of senescence (data not shown). Using the cytosolic Ca^2+^ probe Fura2, we assessed Ca^2+^ contents both in the cytosol and in the intracellular stores (i.e., mainly Ca^2+^ stored in endoplasmic reticulum and mitochondria). The size of intracellular Ca^2+^ stores was appreciated as the Ca^2+^ increase in the cytosolic Ca^2+^ compartment due to the ionomycin-induced membrane permeabilization and the subsequent organelles’ depletion obtained in the absence of external Ca^2+^. All the senescence inducers provoked an increase in the resting cytosolic Ca^2+^ concentration and in the intra-organelle Ca^2+^ content (Figure 1). Therefore, this global Ca^2+^ increase appears to be a mark of cellular senescence in HMECT.

### 2.2. CALB1 Expression Is Induced in Senescent HMECT

**Figure 2:**
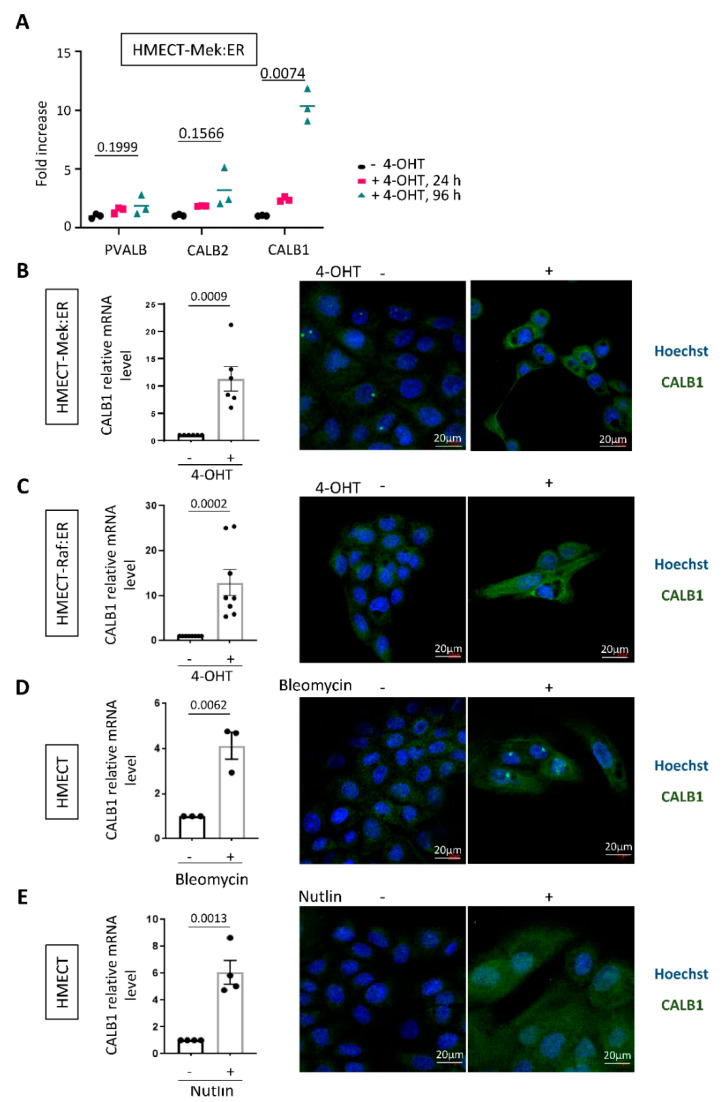
CALB1 expression is increased in HMECT in response to diverse senescence inducers. (**A**) Transcriptomics analysis in HMECT-Mek:ER reveals that CALB1 expression is induced after oncogene activation by 4-OHT. PVALB, CALB2, and CALB1 mean expression level from a biological triplicate is indicated at 0 (−4-OHT), 24 and 96 h after 4-OHT treatment. An unpaired *t*-test with Welch correction was used. (**B**–**E**). CALB1 mRNA level (**left** panels) and protein level and subcellular localization (**right** panels) were assessed in HMECT either overexpressing 4-OHT-inducible oncogenes Mek:ER (**B**) or Raf:ER (**C**) or treated with bleomycin (**D**) or nutlin (**E**). CALB1 mRNA level was measured by RT-qPCR and mean +/- SEM of independent experiments are shown ((**B**), n = 6; (**C**), n = 8; (**D**), n = 3; (**E**), n = 4). Unpaired *t*-test (**B**,**D**,**E**) or Mann–Whitney test (**C**) were performed, and *p*-values are indicated. CALB1 protein level and localization were assessed six days after treatment by immunofluorescence with CALB1 antibody, nuclei were stained with Hoechst.

Ca^2+^ homeostasis is crucial within the cell as a high Ca^2+^ concentration can lead to cell death, whereas a low Ca^2+^ concentration blocks some crucial cellular pathways because Ca^2+^ acts as a cofactor of multiple enzymes and pathways [16]. The fine-tuning of Ca^2+^ concentration in the different cellular compartments relies on various Ca^2+^ channels, pumps, transporters, or calcium-buffering proteins. To identify new Ca^2+^ regulators during oncogene-induced senescence, we explored whole genome transcriptome data that we recently obtained in HMECT-Mek:ER [17] and noticed an induced expression of CALB1 (calbindin 1, also called calbindin-D28K) after Mek activation with 4-OHT (Figure 2A). CALB1 is a calcium-binding protein well known to buffer cytosolic Ca^2+^ contents and thus participate in Ca^2+^ homeostasis [12]. mRNA levels of other Ca^2+^-buffering proteins such as CALB2 or PVALB were not significantly induced by Mek after four days of activation, and S100G mRNA was not detected in HMECT cells (Figure 2A). We then investigated the level and localization of CALB1 in HMECT in the different models of senescence described in Figure 1. We confirmed CALB1 induction by RT-qPCR and immunofluorescence upon Mek-induced senescence in HMECT (Figure 2B). Interestingly, in the other senescence contexts, an increase in CALB1 level was also observed by RT-qPCR and immunofluorescence (Figure 2C–E). CALB1 staining might indicate mainly cytosol localization, as expected, although we cannot rule out other localization (Figure 2B–E). Altogether these results support that senescent HMECT cells display increased Ca^2+^ contents together with increased expression of a calcium-buffering protein, CALB1.

### 2.3. CALB1 Expression Is Induced by the Calcium-Dependent Calcineurin/NFAT Pathway and Its Constitutive Expression Limits Intracellular Ca^2+^ Accumulation upon OIS in HMECT

**Figure 3:**
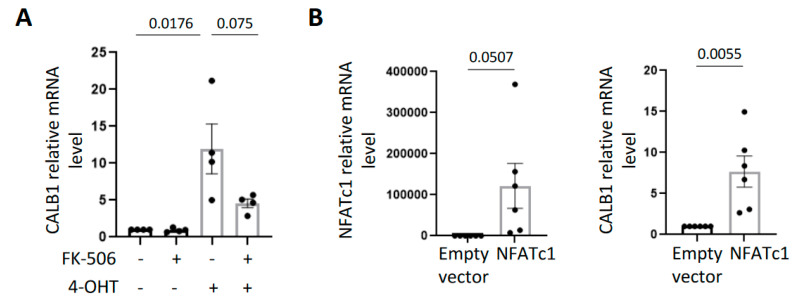
The calcium-dependent calcineurin/NFAT pathway induces CALB1 expression upon oncogene activation in HMECT. (**A**) CALB1 mRNA level was measured by RT-qPCR in HMECT overexpressing 4-OHT-inducible Mek:ER and treated (+) or not (−) with FK-506, a calcineurin inhibitor (treated every day with 2 µM during six days), or/and with 4-OHT (100 nM). The first three days, cells were co-treated with 4-OHT and FK-506 (first treatment 4 h prior 4-OHT), and the three last days only with FK-506. Mean +/− SEM of four independent experiments are shown. An unpaired *t*-test was performed and *p*-value is indicated. (**B**) NFATc1 (**left** panel) and CALB1 (**right** panel) mRNA levels were measured by RT-qPCR in HMECT 24 h after infection with a vector encoding NFATc1 or control empty vector. Mean +/− SEM of six independent experiments are shown. Unpaired *t*-tests were performed, and *p*-values are indicated.

**Figure 4:**
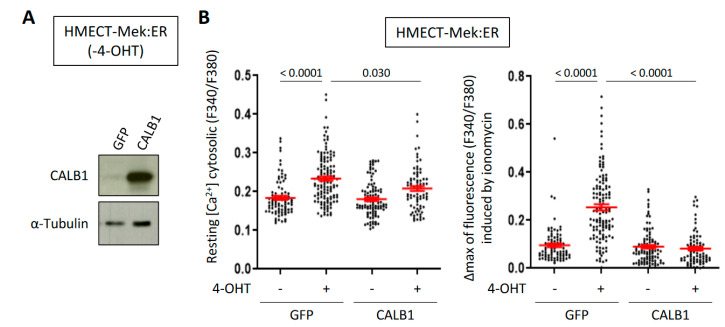
CALB1 overexpression buffers increased Ca^2+^ levels induced by oncogene activation in HMECT. (**A**) CALB1 protein level was assessed by Western Blot in HMECT-Mek:ER (without 4-OHT) infected with a vector encoding CALB1 or the corresponding GFP control vector. α-Tubulin protein level was assessed as a loading control. (**B**) Ca^2+^ levels were measured with the ratiometric probe Fura2 in HMECT-Mek:ER inducible oncogene and CALB1 or the corresponding GFP control vector, with or without 4-OHT treatment. Resting cytosolic Ca^2+^ concentration was evaluated as a stable fluorescent ratio before stimulation (**left** panel), and the size of Ca^2+^ intracellular stocks was estimated as the Ca^2+^ peak amplitude obtained after ionomycin stimulation (**right** panel). Means +/− SEM of 3 independent experiments are presented. Kruskal–Wallis test was performed, and *p*-values are indicated. Three days after 4-OHT treatment, GFP- 4-OHT n = 85 or + 4-OHT n = 126, CALB1- 4-OHT n = 99 or + 4-OHT n = 77.

Ca^2+^ as a secondary messenger is known to activate different calcium-dependent signaling pathways. Notably, it binds to calmodulin, activating calcineurin which allows NFAT transcription factor translocation into the nucleus and activation. NFAT transcription factors have been described to directly induce CALB1 expression in pancreatic β cells [18]. Moreover, pharmacological inhibition of the calcineurin/NFAT pathway was reported to counteract p53-dependent cancer cell senescence [7]. We thus investigated whether the calcium-dependent calcineurin/NFAT pathway could impact CALB1 expression in OIS. First, we treated HMECT-Mek:ER cells with a commonly used chemical inhibitor of calcineurin, FK-506. FK-506 treatment impaired the induction of CALB1 expression upon Mek activation (Figure 3A), indicating the role of NFAT transcription factors in CALB1 induction. This inhibition was not complete, suggesting that other transcription factors than NFAT can contribute to CALB1 induction during OIS. Still, supporting a role of NAFTc1, its overexpression led to an increase in CALB1 mRNA level (Figure 3B). This suggests that upon OIS in HMECT, increased Ca^2+^ content could induce CALB1 expression through NFATc1 activation. As CALB1 is a potent calcium-buffering protein, we next investigated whether CALB1 overexpression could affect Ca^2+^ levels in OIS. We observed that, indeed, CALB1 overexpression (Figure 4A) impairs the increase in Ca^2+^ levels in the cytosol and intracellular stocks during OIS (Figure 4B). Thus, induction of CALB1 during OIS could limit the Ca^2+^ increase in senescent cells.

### 2.4. Increased Expression of Calbindins Is a Frequent Mark of Senescent Cells

**Figure 5:**
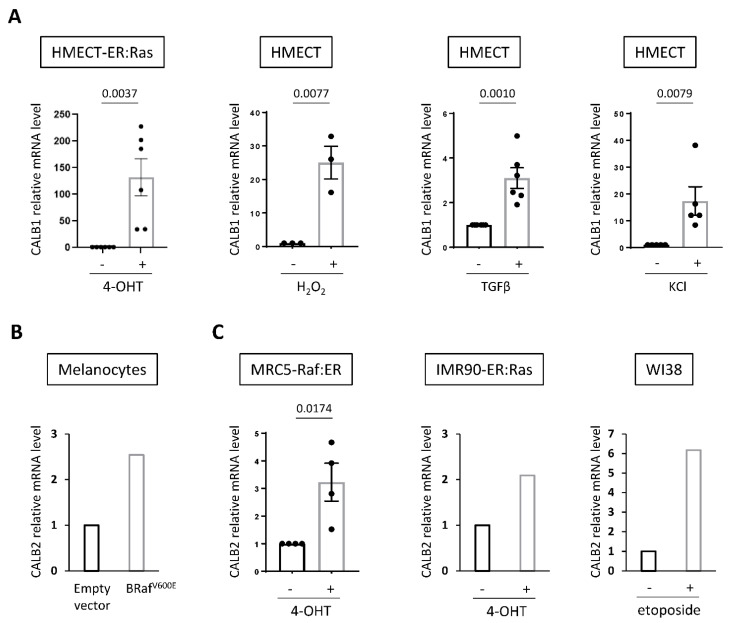
Expression of calbindins is induced in many senescence contexts. (**A**) Additional contexts where CALB1 expression is upregulated in HMECT in response to senescence inducers. CALB1 mRNA level was measured by RT-qPCR in HMECT overexpressing 4-OHT-inducible ER:Ras oncogene (day 6 after 100 nM 4-OHT treatment, n = 6) or treated with H_2_O_2_ (at 250 µM for 1 h and RNA were collected six days later, n = 3), TGFβ (at 0.5 ng/mL for three days, n = 6) or KCl (at 65 mM for 24 h, n = 5). (**B**,**C**) CALB2 expression is increased in several senescence contexts. (**B**) CALB2 mRNA level measured in melanocytes overexpressing B-RAF^V600E^ oncogene (transcriptome data [22]). (**C**) CALB2 mRNA level measured in normal human embryonic lung fibroblasts MRC5 overexpressing 4-OHT-inducible Raf:ER oncogene (RT-qPCR at day 3 after 100 nM 4-OHT treatment, n = 4), IMR90 overexpressing 4-OHT-inducible ER:Ras oncogene (transcriptome data [23]) or WI38 treated with etoposide (transcriptome data [24]). For RT-qPCR, mean +/− SEM of independent experiments are shown. Unpaired *t*-test (for HMECT-ER:Ras, HMEC H_2_O_2_, HMEC TGFβ, and MRC5-Raf:ER) or Mann–Whitney test (for HMECT KCl) were performed and *p*-values are indicated.

Finally, as an increase in intracellular Ca^2+^ contents appears to be a common mark of senescence based on our data in epithelial cells (Figure 1) and on data in other cell types according to previous reports [6,7,11], we investigated whether induction of the expression of calcium-buffering calbindin-type proteins could also be a common mark of senescent cells. In addition to Raf and Mek oncogenes, bleomycin, and nutlin (Figure 1), other senescence inducers such as Ras oncogene activation, H_2_O_2_, TGFβ, or KCl, which are all known to induce senescence in HMECT [14,19,20,21], also triggered a rise in CALB1 expression in HMECT (Figure 5A). We then explored if an induced expression of some calbindins could also be observed upon senescence in other cell types by interrogating published transcriptome data as well as performing RT-qPCR. In melanocytes induced in senescence by B-Raf^V600E^ oncogene, no increase in CALB1 expression could be detected (data not shown), but induction of mRNA levels of CALB2 (calbindin 2, also called calbindin-D29K or calretinin), another well-known calcium-buffering protein (Figure 5B) [22]. CALB2 expression was also increased in normal human fibroblasts upon Raf activation, as well as in response to other senescence-inducing stresses such as Ras activation [23] or etoposide treatment [24] (Figure 5C). Expression of calbindins is thus induced in senescent cells, but induction of both CALB1 and CALB2 in the same senescence model was not observed, suggesting cell-type specificity in the calbindin induced during senescence.

## 3. Discussion

In this study, we showed that intracellular Ca^2+^ levels in the cytosol and in the intracellular stocks rise in HMECT in response to diverse senescence inducers. Moreover, we discovered that mRNA levels of a calcium-buffering protein, CALB1, are increased in these contexts. CALB1 expression is induced by the calcineurin/NFAT pathway, which is activated by Ca^2+^. We further showed that CALB1 overexpression in oncogene-induced senescence buffers the rise in Ca^2+^ levels. Finally, we observed that expression of other calcium-buffering calbindin-type proteins can be upregulated in senescence models where CALB1 is not induced, suggesting that senescent cells have developed strategies to tightly regulate the rise of Ca^2+^ during senescence.

Together with other works which reported increased Ca^2+^ levels in several senescence contexts [5,8,9,10,11], our observations suggest that a rise in intracellular Ca^2+^ contents could be a new hallmark of cellular senescence. Although some contributions of Ca^2+^ in senescence have already been identified, its precise role in this context is not fully understood yet [5,8,9,10,11]. In our study, we observed an increase in Ca^2+^ contents both in the cytosol and in intracellular stocks, which are mainly in the ER and in the mitochondria. Ca^2+^ contained in these two organelles plays important roles in diverse cellular processes such as ATP or reactive oxygen species (ROS) production and contributes to ER stress. We recently showed that during senescence, mitochondrial Ca^2+^ concentration increases notably due to Ca^2+^ transfer from the ER promoted by MERCs. Accumulation of Ca^2+^ in mitochondria induces ROS generation and DNA damage, which lead to cell entry into senescence [8,9,10]. A role of Ca^2+^ in the regulation of SASP has also been reported as the precursor of interleukin 1α (IL1α), a key upstream SASP pro-inflammatory cytokine inducing the expression of other SASP cytokines such as IL6 and IL8, is processed by the calcium-dependent calpain protease [25]. Ca^2+^ also activates mTOR and NLRP3 [26,27], which are instrumental for IL1α translation and processing. Consequently, a rise of Ca^2+^ in senescent cells might contribute to many features of senescent cells [5].

This rise of Ca^2+^ seems to result in the up-regulation of calcium-buffering protein, CALB1, in senescent HMECT. Indeed, according to our results, chemical inhibition of the calcium-dependent calcineurin/NFAT pathway largely inhibits the upregulation of CALB1 in senescent HMECT, whereas constitutive expression of NFATc1 transcription factor activates CALB1 expression in HMECT. In this latter condition, mRNA level of overexpressed NAFTc1 is very high compared to the level of induction of CALB1 messenger. This difference could be explained by a low level of transcriptionally active NFAT protein and/or CALB1 promoter saturation and/or limitation of cofactors. Our findings are in accordance with a previous report describing that NFAT pathway upregulates CALB1 in another context [18]. Importantly, this pathway also induces the expression of ITPR2 ER Ca^2+^ release channel by directly binding to its promoter [28], ITPR2 being a key regulator of cellular senescence [8,9,10]. The calcineurin/NFAT pathway could thus impact senescence by regulating Ca^2+^ signaling at several levels. Up to now, this pathway has been reported either to promote or suppress senescence depending on the cell type and context [7,29], and future studies will be required to better understand the role of the calcineurin/NFAT pathway in regulating Ca^2+^ signaling during cellular senescence.

The role of CALB1 in senescence remains to be explored. It was recently reported that a high level of CALB1 could inhibit senescence induction by promoting p53 degradation through HDM2 activation in cancer cells [30], but Ca^2+^ contribution was not investigated in this study. We showed that CALB1 overexpression in HMECT buffers the rise in intracellular Ca^2+^ contents in oncogene-induced senescence. However, we did not observe in our preliminary experiments any impact of this overexpression on cell proliferation arrest induced by Mek activation (data not shown). CALB1 was reported to protect cells from death by buffering Ca^2+^ [31]. Moreover, CALB1 does not only act as a calcium-buffering protein but also as a Ca^2+^ sensor, interacting in a calcium-dependent manner with other proteins. Notably, after Ca^2+^ fixation to its EF-hands, CALB1 interacts with and inhibits caspase 3, a key effector caspase in the apoptosis process [32]. This inhibition was shown to suppress apoptosis upon CALB1 overexpression in osteoblastic cells [33]. As a too high intracellular Ca^2+^ concentration triggers cell death, one interesting hypothesis would be that CALB1 expression is upregulated in response to senescence-inducing stresses in order to buffer the rise in intracellular Ca^2+^ levels and avoid its cytotoxic effects. This negative feedback loop would be established by the calcium-dependent calcineurin/NFAT pathway. The observation of an increased expression of calbindins in response to many senescence-inducing stresses in several cell types suggests that this could be a general mechanism in senescent cells. This idea will need to be explored by investigating the impact of downregulating the expression of calbindins on senescence.

Altogether, by identifying the induction of the expression of calbindins in senescent cells, our work points out a new feature of senescent cells and provides a better understanding of calcium-related processes in cellular senescence. Here, we focused our work on normal senescent cells, it will also be interesting to investigate whether these mechanisms are shared by senescent cancer cells, as some molecular features they express are different [34]. This work paves the way for future studies investigating new potential molecular basis underlying senescent cell resistance to cell death, which might be exploited for designing new senolytic approaches. 

## 4. Materials and Methods

### 4.1. Cell Culture and Reagents

Human mammary epithelial cells (HMEC, CC-2551B, Lonza, Basel, Switzerland) were cultured in a mammary epithelial cell growth medium (C-21210, Promocell, Heidelberg, Germany) supplemented with 1% penicillin/streptomycin (Life Technologies, Carlsbad, CA, USA). Human fetal lung fibroblasts MRC5 (ATCC), 293 GP retrovirus-producing cells, and 293 T lentivirus-producing cells (Clontech, Mountain View, CA, USA) were cultured in Dulbecco’s modified Eagle’s medium (DMEM supplemented in GlutaMax, Life Technologies) with 10% fetal bovine serum (FBS, Sigma-Aldrich, St. Louis, MO, USA) and 1% penicillin/streptomycin (ThermoFisher Scientific, Waltham, MA, USA). All the cells were cultured at 37 °C with 5% CO_2_. (Z)-4-hydroxytamoxifen (4-OHT) (Sigma-Aldrich) was used at 100 nM, bleomycin (Merck, Kenilworth, NJ, USA) at 12 µg/mL, nutlin-3 (Sigma-Aldrich) at 1 µM, H_2_O_2_ (Sigma) at 250 µM, TGFβ (Peprotech, Rocky Hill, NJ, USA) at 0.5 ng/mL, KCl (Sigma-Aldrich) at 65 mM and FK-506 (MedChem Express, Monmouth Junction, NJ, USA) at 2 µM.

### 4.2. Vectors, Transfection, and Infection

HMEC were first infected with pWZL-Blast-Flag-HA-hTERT retroviral vector [35] (Addgene_22396) encoding the catalytic subunit of telomerase to generate immortalized HMEC (HMECT). HMECT or MRC5 were infected with pLNC-Mek:ER (ΔMEK1 (ΔN3,S218E, S222D):ER) [9], pBabe-Raf:ER (HA-∆Raf1(S642A):ER) [36] (Addgene_72572) or pLNCX-ER:Ras (ER1a H-RasG12V) [37] (Addgene_67844) retroviral vectors encoding Mek, Raf or Ras oncogenes respectively, fused to the ligand binding domain of estrogen receptor (ER). Upon 4-OHT treatment, these ER-fused oncogenes are stabilized and activated [9,36,37]. A retroviral vector encoding a constitutively active NFATc1 ([38], Addgene_11102) and a lentiviral vector encoding CALB1 (pLV-CALB1, VectorBuilder) were used, as well as the corresponding control vectors (empty or encoding the GFP). 293 GP retrovirus-producing cells and 293 T lentivirus-producing cells were transfected with GeneJuice transfection reagent (Merck Millipore) according to the manufacturer’s recommendations. Two days after transfection, the virus-containing supernatant was filtered, diluted with fresh mammary epithelial cell growth medium (for HMEC and HMECT) or with fresh DMEM (for MRC5) (1/2 dilution for 293 GP cells and 1/5 dilution for 293 T cells) and hexadimethrine bromide at 8 µg/mL (Sigma-Aldrich) and used to infect HMEC/HMECT or MRC5. On the following day, media were changed, and selection of infected cells was started using puromycin (500 ng/mL; Invivogen) or geneticin (100 μg/mL; Life technologies). HMEC and HMECT are estrogen-independent cells, and accordingly, they do not respond to 4-OHT at the concentration used. The generated HMECT expressing inducible oncogenes were used within the next 2–4 weeks to avoid selection of specific clones or problems of ploidy.

### 4.3. RNA Extraction, Reverse Transcription and Real-Time Quantitative PCR

Total RNAs were extracted using NucleoZOL (Macherey-Nagel) according to the manufacturer’s instructions. RNAs were reverse transcribed with First-Strand cDNA Synthesis Kit (GE Healthcare) following the manufacturer’s recommendations. TaqMan real-time quantitative PCR was then run with PCR mixture containing TaqMan mix (Roche), 200 nM of primers, Universal Probe Library probe (100 µM, ThermoFisher Scientific), and cDNA template. Reactions were performed in triplicate on a FX96 Thermocycler (Bio-Rad). The comparative Ct (ΔΔCT) method and normalization with GAPDH housekeeping gene were used to calculate the relative amount of mRNA. Sequences of primers are as follows: CALB1:Sens 5′-aagatccgttcggtacagctt-3′, Anti-sens 5′-ctgaaggatctgtgcgagaa-3′; CALB2:Sens 5′-tcatttcctttttgtttttctcg-3′, Anti-sens 5′-gcgatcttcacattttacgaca-3′; NFATc1: Sens 5′-ggtcagttttcgcttccatc-3′, Anti-sens 5′-ccaaggtcattttcgtggag-3′; GAPDH: Sens 5′-agccacatcgctcagacac-3′, Anti-sens 5′-gcccaatacgaccaaatcc-3′.

### 4.4. Western Blot

Cells were lysed in 6X Laemmli buffer (Tris 125 mM pH 6.8, 2% SDS, 10% glycerol) with 15% β-mercaptoethanol and boiled for 5 min at 100 °C. After measuring protein concentrations with Bio-Rad Protein Assay kit according to the manufacturer’s recommendations, total cell extracts were separated using 12% acrylamide gel by SDS-PAGE electrophoresis, and proteins were transferred to nitrocellulose membranes (Bio-Rad, Hercules, CA, USA). Membranes were blocked for 1 h in Tris buffer saline (TBS, pH 7.5), with 0.05% Tween-20 (TBS-T) and 5% milk and primary antibodies added and incubated overnight at 4 °C. An anti-CALB1 rabbit polyclonal antibody (sc28285, Santa Cruz, Santa Cruz, CA, USA) and anti-α-tubulin mouse monoclonal antibody (T6199, Sigma-Aldrich) were used. After washes in TBS-T, membranes were incubated for 1 h at room temperature with anti-rabbit (711-035-152, Interchim, Montluçon, France) or anti-mouse (715-035-150, Interchim) HRP-coupled secondary antibody (1/5000 dilution). Peroxidase activity was revealed using an enhanced chemiluminescence Western Blotting detection reagents (RPN2106V1/2, GE Healthcare, Chicago, IL, USA) and visualized by luminography.

### 4.5. Immunofluorescence Staining

Cells plated on Lab-Tek chamber slides (Thermo Fisher) were washed once with PBS and fixed with ice-cold methanol 10 min at −20 °C. Cells were then rinsed once in TBS-T and incubated in TBS-T-20% FBS for 30 min at room temperature. Cells were then incubated overnight at 4 °C with primary antibody against CALB1 (sc28285, Santa Cruz). The following day, cells were washed and incubated with anti-rabbit IgG coupled with Alexa Fluor 488 (A11008, Life Technologies) for 1 h at room temperature. Cells were then washed with PBS and counterstained with Hoechst (Sigma-Aldrich) for 10 min at room temperature. Pictures were taken with a Nikon fluorescence microscope (CRCL cellular imaging platform).

### 4.6. Calcium Imaging

Cytosolic Ca^2+^ experiments were achieved as previously described [39]. Briefly, plated cells were loaded with the Fura2-AM dye (2.5 µM; Molecular Probes) for 30 min at 37 °C in Ca^2+^-containing buffer (HBSS; Gibco). Live Ca^2+^ imaging was performed at room temperature and in the absence of external Ca^2+^ (Ca^2+^-free HBSS; Gibco). MetaFluor 6.3 (Universal Imaging) was used for image analysis. After 2 min at resting state, cells were stimulated with 1 µM ionomycin in order to deplete intracellular Ca^2+^ stores. Results are presented as a ratio of fluorescence intensity of 340/380 nm (F340/F380) as this ratio is strictly related to intracellular free calcium [40,41].

### 4.7. Statistical Analyses

Graphs show means and SEM obtained with several independent experiments (n indicated in figure legends) or show data extracted from transcriptome analyses as mentioned in figure legends. Statistical analyses were performed with GraphPad Prism 8 software and are specified in figure legends. Before proceeding to any analysis, the normality of the samples was evaluated. Unpaired *t*-test (for normal distribution) or Mann–Whitney test (for non-normal distribution) was used unless stated otherwise in the figure legends. *p*-values are indicated in figures.

## Data Availability

The data supporting findings presented in this study are available from the corresponding author upon reasonable request.
